# Posterior spinal decompression in adults with spinal cord injury without traumatic compromise of the spinal canal: what is the data?

**DOI:** 10.3389/fneur.2023.1220598

**Published:** 2023-09-15

**Authors:** Mary Solou, Anastasios A. Politis, Ιoannis Ydreos, Evangelos K. Papadopoulos, Stamatios Banos, Georgios Savvanis, Maria M. Gavra, Efstathios J. Boviatsis, Lampis C. Stavrinou

**Affiliations:** ^1^Department of Neurosurgery and Neurotraumatology, “Attikon” University General Hospital, National and Kapodistrian University of Athens, Athens Medical School, Athens, Greece; ^2^Department of CT and MRI Imaging, “Agia Sofia” Children’s Hospital, Athens, Greece

**Keywords:** spinal cord injury (SCI), SCI, laminectomy, laminoplasty, durotomy, duroplasty

## Abstract

**Background:**

Spinal cord injury (SCI) can be caused by a variety of factors and its severity can range from a mild concussion to a complete severing of the spinal cord. Τreatment depends on the type and severity of injury, the patient’s age and overall health. Reduction of dislocated or fractured vertebrae via closed manipulation or surgical procedures, fixation and removal of bony fragments and debris that compromise the spinal canal are indicated for decompression of the spinal cord and stabilization of the spine. However, when there is no obvious traumatic obstruction of spinal canal, the question arises as to whether laminectomy is needed to be performed to improve neurological outcome.

**Methods:**

A literature review covering all indexed studies published between 2013 and 2023 was performed using keywords to identify the patient group of interest (spinal cord injury, SCI, spinal cord trauma, cervical, thoracic, lumbar, thoracolumbar),central cord syndrome (CCS) and the interventions (laminectomy, laminoplasty, decompression, duroplasty).

**Results:**

This review includes6 observational studies investigating the outcome of posterior spinal decompression in patients suffering from spinal cord injury without traumatic spinal cord stenosis. Most patients already had degenerative stenosis. From a total of 202, 151 patients (74.7%) improved neurologically by at least one grade at ASIA scale, after being treated with either laminectomy, laminoplasty, duroplasty or a combination of these techniques.

**Conclusion:**

Early decompression in SCI patients remains a reasonable practice option and can be performed safely, but no specific evidence supports the use of laminectomy alone. There is emerging evidence that intended durotomy followed by extended meningoplasty may improve the neurological outcome in patients suffering from SCI when meta-traumatic edema is apparent. However, the lack of high-quality evidence and results support the need for further research.

## Introduction

Spinal cord injury (SCI) can be caused by a variety of mechanisms, such as falls from height, car accidents, sports injuries, or violence, and has a devastating impact on the quality of life of the affected patients and their caregivers. The severity of the injury can range from a mild concussion to a complete severing of the spinal cord with an increased risk of permanent disability and considerable socio-economic impact (1). Studies have shown that timely and safe transport and diagnosis by healthcare professionals in the acute post-traumatic phase can reduce morbidity and mortality significantly (2). Improving the neurological and clinical outcome remains a challenge and timely treatment seems to be critical (3). Critical care management is based on intensive hemodynamic monitoring and spine immobilization (1). It seems logical and apparent to recommend surgery in cases of spinal instability or persistent spinal cord compression (4). Reduction to restore the spinal alignment may be performed via closed manipulation or surgically, depending on the type of injury. Additional decompression to relieve the pressure on the spinal cord may be needed, by removing any bony fragments and debris that have entered the spinal canal. On the other hand, there are cases of spinal cord injury without fracture or dislocation apparent in imaging scans. This is usually seen in young and middle-aged trauma patients (1, 5). In cases of cervical SCI, this kind of population accounts for about 45% of all cases. Central cord syndrome (CCS) is the most common type of incomplete spinal cord injury. The common setting of CCS without apparent osteoligamentous injury appears to be the closest analog of SCI without compromise of the spinal canal in adults (6). After the primary insult, a plethora of secondary events occur, with progressive edema and hemorrhage predominating and possibly being the cause of spinal cord compression (5, 6). Treatment of such patients may be surgical or conservative depending on the type, the severity of injury, and the clinical status of the patient (7, 8). For instance, in cervical spine injury without bone or disk involvement, some advocate conservative treatment while others feel a more aggressive approach is warranted (9). Consensus on the management of SCI-free of fractures and spine dislocation has not been established yet.

The majority of studies examine the importance of early decompression after traumatic spinal cord injury, comparing surgical intervention in different time frames in an attempt to identify the optimal time for surgical intervention. Early decompression and stabilization seem to yield the best results, but the decision should be individualized (3, 7). While there are various studies examining the effect of decompression after SCI in different animal models that have shown promising neurological results, little evidence exists on the management of patients with SCI without obvious obstruction of the spinal canal.

Surgical decompression aims to relieve the cause of the compression, thereby reducing secondary hypoxia and ischemia, potentially resulting in a better neurological outcome (3, 5). While arguing on the safety and effectiveness of decompression surgery, a specific question arises as to whether laminectomy is needed to improve neurological outcome in such SCI patients free of spinal obstruction. The purpose of this study is to review the literature on studies investigating the management of adult SCI patients without apparent traumatic stenosis of the spinal canal and to evaluate whether laminectomy is beneficial for improving the neurological outcome in such cases.

## Methods

To examine the effect of laminectomy on neurological outcome following spinal cord injury after closed or surgical reduction and without any obvious traumatic obstruction of the spinal canal, a literature review was performed. This manuscript was structured in accordance with “The Preferred Reporting Items for Systematic Reviews and Meta-Analysis (PRISMA) guidelines” [Supplementary material 1, (10)].

### Information sources and search strategy

Articles published between January 2013 and March 2023 regarding decompression of the spinal canal in patients with SCI without traumatic compromise of the spinal canal were reviewed using the Pubmed search engine. The following keywords were used to identify this patient group (Supplementary material 2): spinal cord injury, SCI, spinal cord trauma, cervical, thoracic, lumbar, thoracolumbar, central cord syndrome, SCIWORA (Spinal Cord Injury without Radiographic Abnormality), and the interventions (laminectomy, laminoplasty, decompression, duroplasty/meningoplasty, and durotomy). The reference lists of the selected articles were also reviewed.

### Eligibility criteria

Included were studies (i) written in English, (ii) had an abstract and full text available, (iii) with a specific study design (meta-analysis, systematic review, randomized controlled study, prospective, and retrospective cohort study, and review-type studies) and referring to a (iv) population of adults (defined as age ≥ 18 years old), (v) undergoing decompression of spinal cord for SCI after being treated with closed or surgical reduction, and (vi) without any obvious traumatic stenosis of the spinal canal -no vertebrae fracture or dislocation compressing the spinal cord. Patients with prior degenerative spinal canal stenosis were included. From the study were excluded studies (i) referring to spine injury with traumatic canal stenosis, (ii) with only abstract available, (iii) animal and lab studies, (iv) case reports, (v) pediatric population studies, (vi) papers published in other than English language, and (vii) published prior to 2013.

### Selection and data collection process

The selection of study items followed a double-reviewer process. The records identified in Pubmed using date filters (01/2013–03/2023) and the foretold keywords were checked for duplicates. Then, articles were screened according to the selection criteria mentioned above using Rayyan online platform (11). The retrieved papers were examined in full for eligibility. The final list of articles included some studies from the respective reference lists. After articles were selected for inclusion, we reviewed the characteristics of each study and collected the relevant data.

### Data items, definitions, and outcomes

Laminectomy is a surgical procedure that creates space in the spinal canal by removing part or all of the lamina of one or more spine vertebrae. Laminoplasty describes the surgical technique of enlarging the spinal canal by reconstructing the lamina arch and thus propping open the spinal canal. In the context of this review, durotomy is used in conjunction with duroplasty and describes the process of expanding the spinal intradural space by placing or stitching an (autologous or artificial) patch to an opened dura. SCIWORA is the short form of “Spinal Cord Injury without Radiographic Abnormality,” defining the patients experiencing symptoms of posttraumatic spinal cord injury without radiological evidence of fracture or spine malalignment. By referring to traumatic compromise of the spinal cord, we mean a spinal cord injury that causes compression to the spinal cord.

The neurological outcome measured in the ASIA scale after decompression of the spinal canal for SCI was considered as the primary outcome (12). Secondary outcomes included morbidity, mortality, and postoperative complications. All included studies were assessed for the level of evidence and quality, then conflicts were discussed and resolved.

### Study synthesis

The literature review was structured by analyzing the data from the included studies, comparing the homogenous data between them, and using the online statistics calculator “DATATab” [DATAtab Team (2023). DATAtab: Online Statistics Calculator. DATAtabe.U. Graz, Austria. URL https://datatab.net] for data analysis (13). Tables were created to illustrate more specifically the results of this systematic review. Microsoft® Excel was used for chart making (Microsoft Corporation. Microsoft Excel, 2018. Available from: https://office.microsoft.com/excel).

## Results

**Figure 1:**
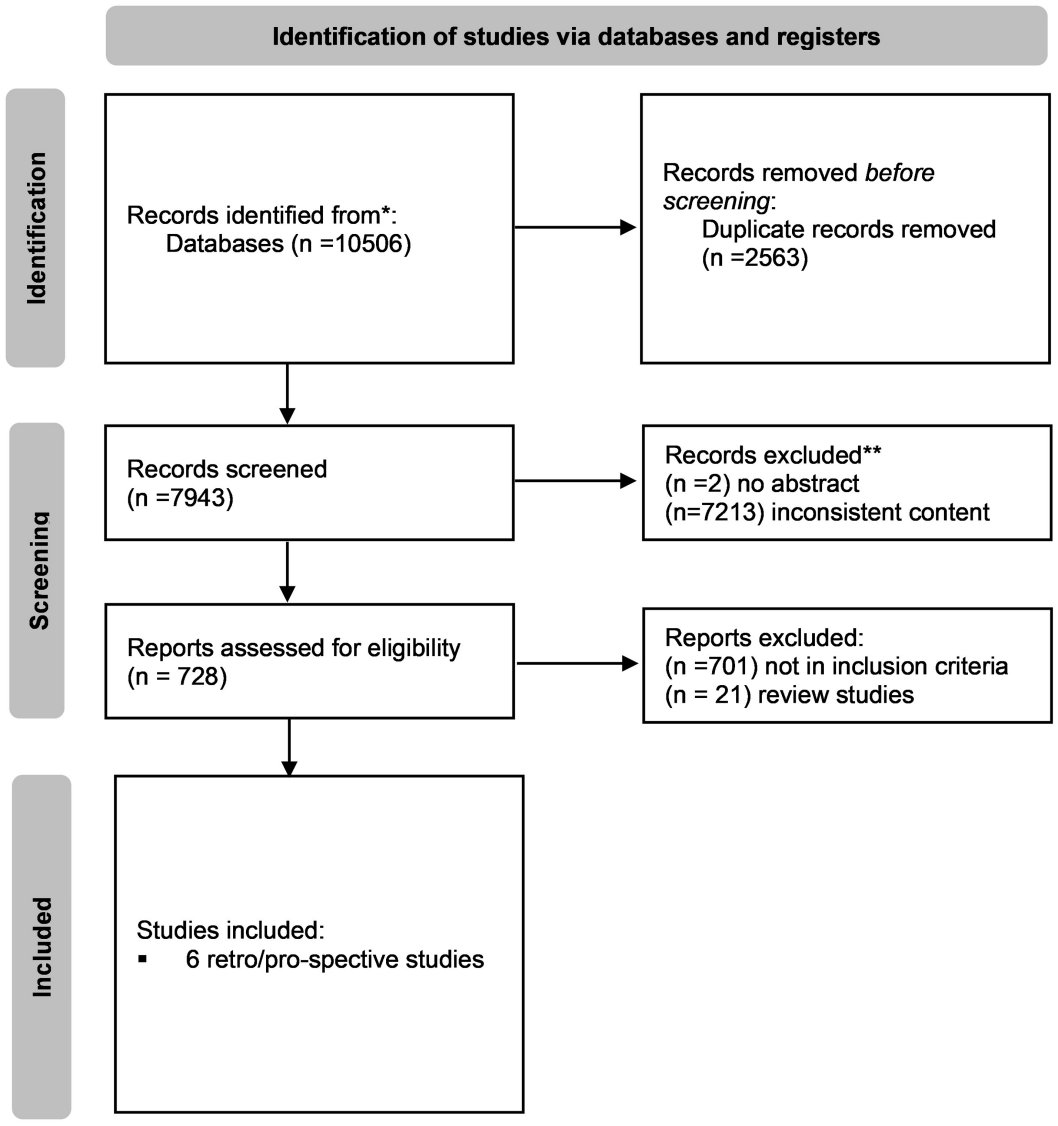
“PRISMA flowchart of selection process” (10).

The search identified 10,506 studies, of which 2,563 were duplicates. From the 7,943 records screened on a title-abstract basis, 2 reports could not be retrieved and 7,213 studies were excluded due to inconsistent research content. From 728 full-text studies being left for eligibility assessment, 722 records not following the inclusion criteria were excluded after a double-reviewer process. In total, this systematic review included 6 observational retrospective or prospective studies (Figure 1).

### Data of included studies

In this study, data were derived from 6 observational studies investigating the neurological outcome after decompression of the spinal cord in patients with SCI without obvious traumatic spinal canal stenosis – be it fracture, vertebrae dislocation, or disk rupture. The main demographics of the included studies are shown in Table 1. The included studies either investigate the outcome in patients with SCI after laminectomy/laminoplasty/durotomy/duroplasty, or compare such treatment methods between them or to conservative management. In one study, Phang et al., which compares laminectomy alone (intervention arm) to laminectomy with duroplasty (control arm), both patient groups were eligible for our study and therefore we have added both intervention and control arms as separate groups in our study (14). Therefore, Table 1 consists of 7 groups of patients with SCI without traumatic spinal canal stenosis who underwent decompression. While none of the patients included in the studies had obvious traumatic spinal canal stenosis, the majority of the studied population had some degree of degenerative-type spinal stenosis (degenerative intervertebral disk protrusion, spondylosis, ossified posterior longitudinal ligament, etc.).

**Table 1:** Main data and demographics of the studies reviewed.

Author-date	Intervention	Population	Level of injury	No of patients	Average age	Pre-op ASIA grade					Degenerative	Last follow-up (in months)	Patients with improved ASIA (at least 1 grade)
						A	B	C	D	E			
Zhu (5)	Posterior laminectomy followed by durotomy with duroplasty	ASCIWORA (adults with SCI without radiographic abnormality)	cervical	16	47,5	1	12	3	0	0	14	18	16 (100%)
Mazaki (9)	Laminoplasty compared to conservative treatment	Patients with cervical SCI without bone or disk injury	cervical	11	62	3	0	8	0	0	8	3	5 (45.4%)
Jia (6)	Posterior cervical laminectomy compared to anterior cervical discectomy-fusion	Patients with ACCS without fracture or dislocation of the cervical spine	Traumatic cervical central cord syndrome	84	30,19	13	24	34	12	0	84	12	73 (86.9%)
Phang (I) intervention arm (14)	Laminectomy alone (I) compared toduroplasty (II)	Patients with acute severe TSCI with re-alignment of the fracture and surgical fixation	cervical-thoracic	11	43	9	0	2	0	0	NA	25,8	2 (18.8%)
Phang (II) control arm (14)	Laminectomy and duroplasty(II) compared to laminectomy alone (I)	Patients with acute severe TSCI with re-alignment of the fracture and surgical fixation	cervical-thoracic	10	43	7	2	1	0	0	NA	9,4	5 (50%)
Ghasemi (8)	Laminoplasty	Patients with cervical SCI but stable spine	cervical	41	52,1	4	10	17	10	0	41	12	33 (80.4%)
Lee (1)	Laminoplasty	Patients with SCI without instability -spinal cord contusion in MRI	NA	29	57	3	7	9	10	0	29	6	17 (58.6%)
			TOTAL:	202	47.83 ± 10.5y.o	A	B	C	D	E	≈ 176	12.31 m	151
					Range: 19–82y.o	40	55	74	32	0		Range: 3–25.8 m	= (74.7%)

### Result analysis

**Figure 2:**
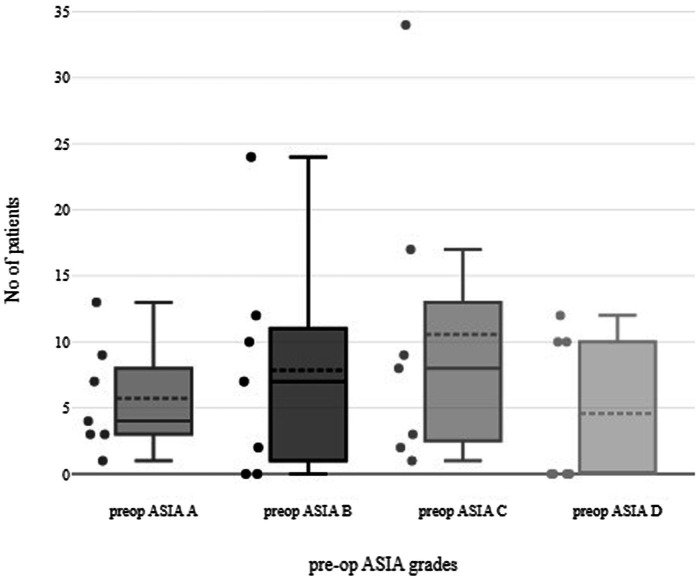
Box-plot representing the pre-op ASIA grade of included patients.

Our review included 202 patients from 6 observational studies. Regarding the level of injury, 5 out of 6 studies are investigating decompression techniques in cervical SCI, while one of them also incorporates patients with trauma of the thoracic spine. One study did not define the level of injury of its population. The included patients were aged 19 to 82 years old, with an average age of 47.83 ± 10.5 years. The preoperative neurological status graded in the ASIA scale was allocated as follows: 40 patients in the ASIA-A group and 55 cases in ASIA-B. The majority of patients (74 cases) belonged to the ASIA-C group and 32 patients to ASIA D or E (Figure 2). As mentioned before, most patients had degenerative stenosis, accounting for at least 176 out of 202 cases. The follow-up time varied from 3 months to 25.8 months, with an average time to be almost a year after surgery, at 12.31 months.

**Figure 3:**
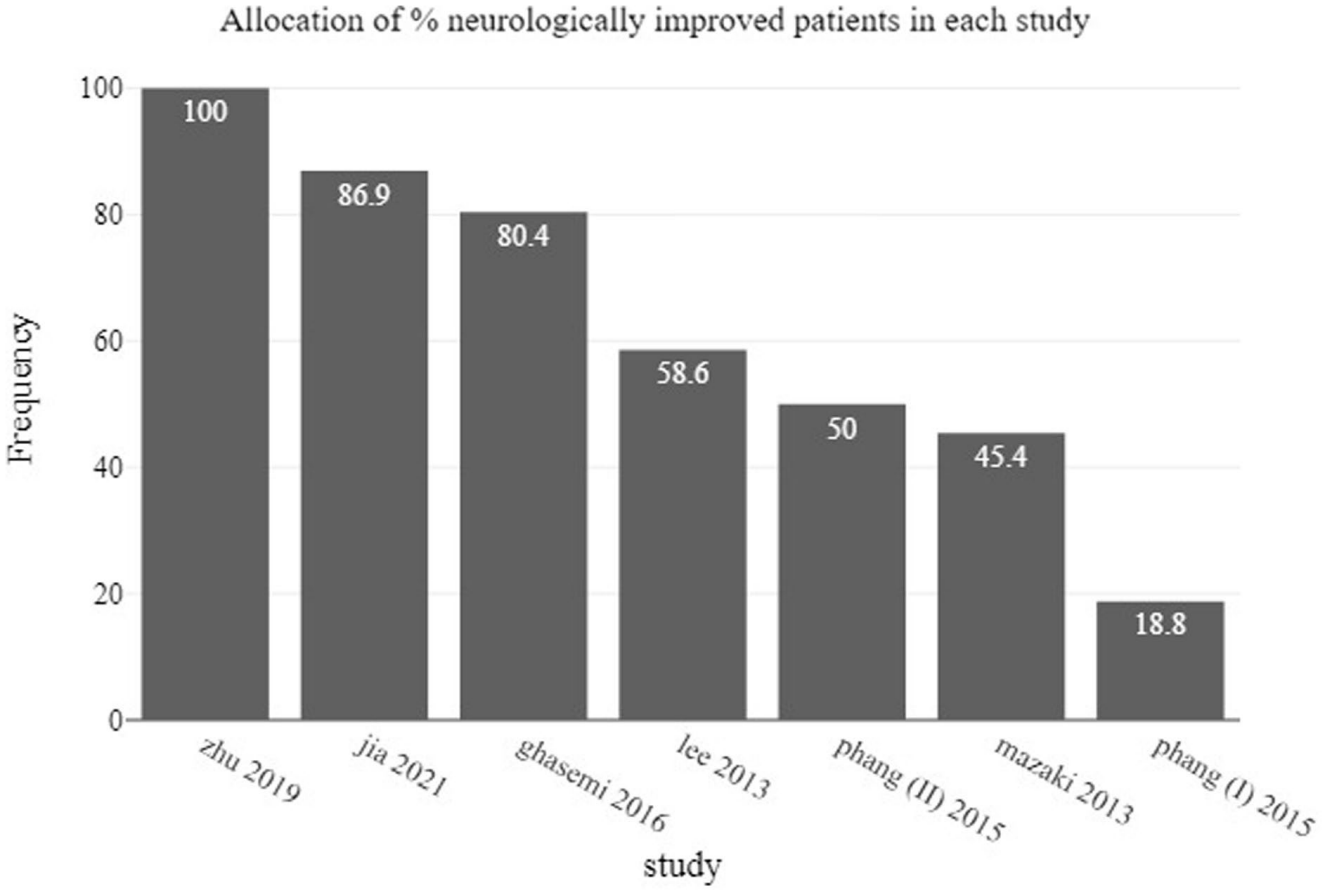
Barchart showing the percentage of neurologically improved patients as allocated in studied groups.

**Figure 4:**
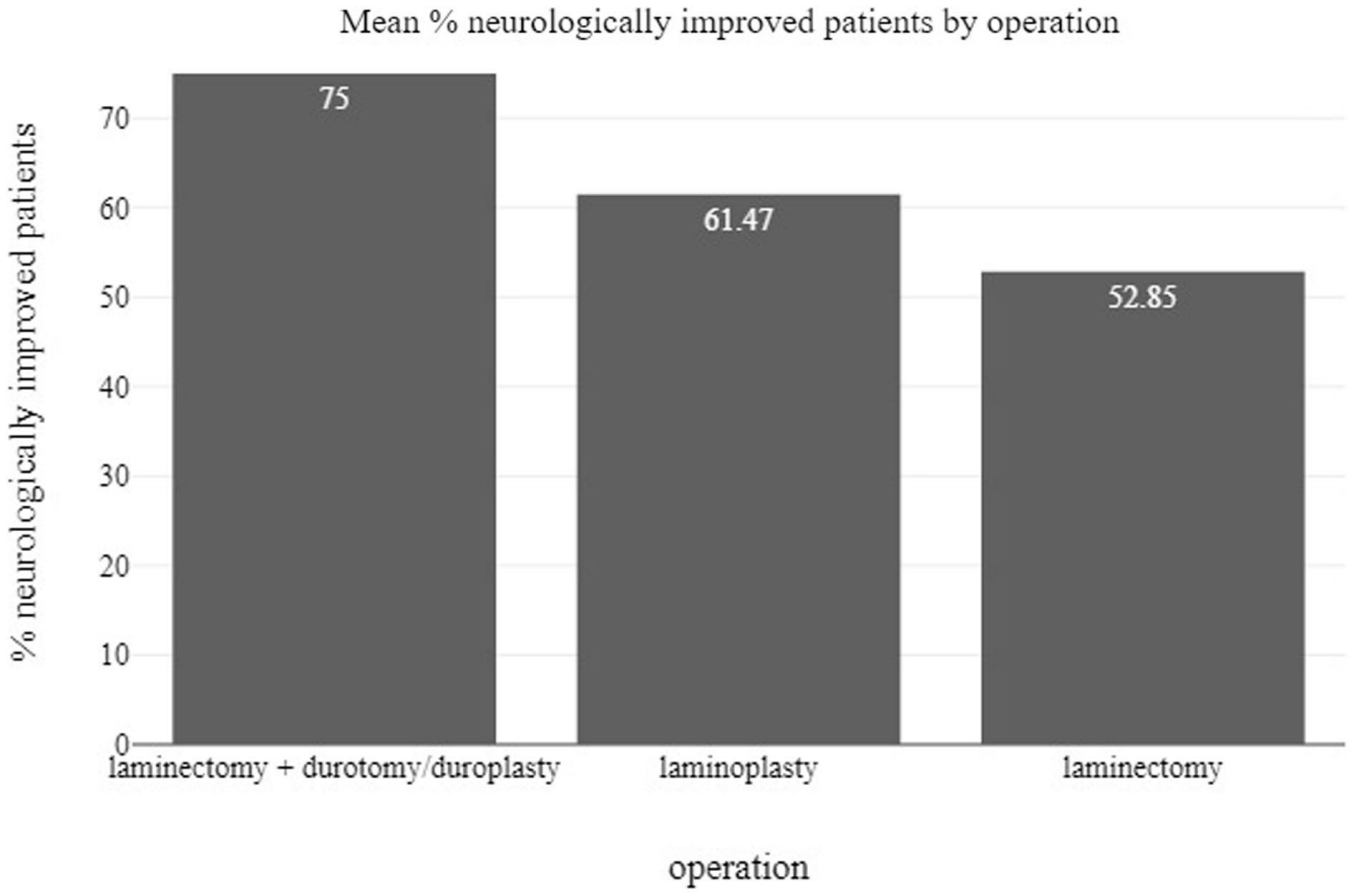
Bar chart illustrating the percentage of patient improvement based on the operation type.

Neurological improvement as measured by the change in the ASIA grade from the preoperative assessment to the final follow-up visit for the entire patient cohort is presented in Table 1. From a total of 202, 151 patients (74.7%) improved neurologically (at least one grade change in ASIA scale) after being decompressed with one of the techniques of either laminectomy, laminoplasty or duroplasty. In Figure 3, the percentage of neurologically improved patients is illustrated in relation to the studied groups. In 2 out of 7 studies less than 50% of the patients improved neurologically. These are the group of Mazaki et al., treated with laminoplasty and being compared to conservative management, and the group of laminectomy alone of Phang et al. which was compared to a group receiving laminectomy and duroplasty. All other studies show improvement of 50% and more in their included patients. Moreover, Figure 4 presents the ratio of patient improvement based on the operation type as it is categorized into three groups regarding the type of decompression: (1) surgical techniques involving the opening of both lamina and dura, (2) decompression via laminoplasty, and (3) when surgical decompression was limited to laminectomy. Greater improvement is seen when bone decompression is followed by dura opening. The main outcomes of the 6 included studies are presented in Table 2.

**Table 2:** Main outcomes of the studies included in our review.

Study	Type of study	Intervention	Outcome
Zhu (5)	R.C-C	Early (<72 h) posterior laminectomy followed by durotomy with duroplasty decompression	Higher AIS score in all patients High level of ISP after laminectomy, whereas ISP continued to decrease steadily after durotomy
Mazaki (9)	P.C-C	Laminoplasty compared to conservative treatment	No significant difference in paralysis improvement between surgical and conservative treatment Higher frequency of complication with surgery
Jia (6)	R.coh	Anterior cervical discectomy-fusion compared to posterior cervical laminectomy	Both anterior cervical discectomy and fusion and posterior cervical laminectomy can improve similarly the ASIA classification −84,52% satisfaction after anterior approach and 86,90% after posterior laminectomy
Phang (14)	P.coh	laminectomy alone compared to laminectomy and duroplasty	Both methods improve ASIA grade but the study was unpowered to reveal significant differences in functional outcomes. Duroplasty improves radiological and physiological parameters No neurological improvement in complete thoracic injury (ASIA A), while 2/3 of complete cervical injury recovered substantially.
Ghasemi (8)	R.C-C	Laminoplasty	Surgical decompression (laminoplasty) is helpful in relieving cord compromise and neurological deficit in CSCI with stable spine Better neurologic outcome in cases with <50% compression of spinal cord
Lee (1)	R.C-C	Laminoplasty	Laminoplasty provided good neurologic recovery in patients with SCI without instability who had cervical canal stenosis, especially due to cervical spondylosis without ossified PLL Better outcome when pre-op ASIA grade is B,C,D

R., retrospective; C-C, case–control; P., prospective; Coh., cohort; ISP, intra-spinal pressure; CSCI, cervical spinal cord injury; PLL, posterior longitudinal ligament.

## Discussion

This study included 202 patients from 6 observational studies divided in 7 groups and showed a trend towards a beneficial effect of decompression of the spinal cord in patients suffering from SCI, especially in cervical spine, even in the setting of an uncompromised spinal canal. Nevertheless, the literature is still inconclusive and one should be cautious before formulating practice recommendations.

The role of the spinal meninges in spinal cord trauma is not fully understood. Certain assumptions derive from the management of traumatic brain injury (TBI): the composition of cell types in the brain parenchyma and the spinal cord is similar and thus they share common pathogenetic mechanisms in an injury. It is well known that after brain injury, the brain swells against the non-expansile dura and skull, with ICP to be increased when the compensatory mechanisms fail. Spinal cord swelling against the dura after SCI may be blamed for the compression by raising the intra-spinal pressure (ISP) and reducing the perfusion of spinal cord (15). Many describe the compression of spinal cord in SCI without bone involvement as a “compartment-like” syndrome caused by the restriction of the cord within the dura. Therefore, removing bony and dural restrictions through laminectomy/laminoplasty and expansion duroplasty in cases of SCI is regarded as the decompressive craniectomy for TBI (15, 16). Along the same line, monitoring has been evolved significantly lately and thus the intra-spinal pressure may be recorded by implanting an intradural extramedullary pressure transducer at the injury site. This seems to be of use in the decision-making for the management of SCI (15).

### Laminectomy-laminoplasty

Ghasemi et al. and Lee et al. studied the effect of laminoplasty and both studies concluded that laminoplasty offers neurological improvement in patients with SCI with uncompromised spinal canal (1, 8). The authors report that better outcome is observed in patients having less than 50% compression of the spinal cord due to degenerative causes (8). In their review, Zhu et al. state that adequate decompression of the spinal cord in reasonable timing can eliminate the associated secondary injury, especially in patients with incomplete injury (3). Additionally, in cases of acute traumatic central cord syndrome (ATCCS), previous studies advocated management similar to cervical myelopathy, with emergent surgery to be proposed in cases of progressive neurological deficits or moderate–severe ATCCS (17). Adequately decompressed patients with ATCCS are reported to have a better prognosis (18). Surgical decompression acts also beneficially to prevent repetitive trauma to the spinal cord by secondary mechanisms which may have led to the deterioration (19). Malliqaj et al. state that there is class III evidence suggesting no difference in outcome between anterior or posterior approach. Similarly, laminoplasty and laminectomy appear equivalent as far as neurological outcome is concerned (17). In the study of Jia et al. comparing 164 patients with acute central cord syndrome who underwent either the anterior cervical discectomy or posterior cervical laminectomy, the authors found similar results between the two approaches, with a slight superiority of the laminectomy technique (6). A more recent retrospective study by Yang et al. regarding the management of patients with SCI also suggests that traumatic edema of the spinal cord is better resolved when an early laminoplasty is performed (20). Lastly, thoracic and lumbar spinal cord injury has not been adequately discussed in the literature and the relation between laminectomy/laminoplasty and neurological outcome is unknown (21).

### Bony decompression followed by durotomy-duroplasty

Literature suggests that in many cases of spinal cord injury, the compression cause involves the dura, hence decompression of the bony spinal canal alone may not alleviate the pressure on the injured spinal cord (22). Saadoun et al. suggest that this happens in more than a quarter of patients. They investigated 21 patients and concluded that compared to laminectomy alone, bony decompression followed by duroplasty adequately widens the dural tube, relieves the intraspinal pressure, and improves cord perfusion, thus improving neurological outcome (22). This could offer a possible explanation as to why studies with only bony decompression have not convincingly benefited the outcome (14). Similarly, the study of Phang et al., with the privilege of comparing laminectomy-alone versus laminectomy followed by duroplasty, showed that both methods can improve the ASIA score in SCI patients, but duroplasty gives better results in radiological and physiological parameters by expanding the space around the injured spinal cord, reducing ISP (intra-spinal pressure) and increasing SCPP (spinal cord perfusion pressure) (14). Being one of the few studies which included patients with thoracic SCI, it is important to mention that in complete *thoracic* injury cases (ASIA A), the decompression did not result in any improvement, while some of the patients with complete *cervical* injury appeared to recover substantially after decompression of spinal cord (14). In their review article, Grassner et al. recommend early surgical decompression and blood pressure augmentation after traumatic SCI. The authors argue that increased intraspinal pressure should be estimated as well, in line with the most recent studies that support a beneficial effect of intentional durotomy in elevating the perfusion pressure of the spinal cord (23). Grassner et al. also note that in small duroplasties, the swollen spinal cord can be restricted at the dural edges while the edema expands, similar to an inadequate craniectomy. On the other hand, larger duroplasties are more commonly associated with complications and require wider laminectomies and potentially fusion procedures (23). Complications may be CSF leakage, pseudomeningoceles, and increased hospitalization time (23). Αcurrently running RCT termed DISCUS (Duroplasty for Injured cervical Spinal Cord with Uncontrolled Swelling) aims to test whether expansion duroplasty after bony decompression improves outcome compared with bony decompression alone in patients with cervical SCI (15).

### Conservative management

A number of studies advocate conservative treatment, reserving surgery for cases with instability and obvious compression of the spinal cord (24–26). Atesok et al. support that cases of SCI with normal MRI scans or intraneural findings like edema or contusion -but without compression- should be managed conservatively (27). Surgery is reserved for patients with ligamentous injury, instability, or spinal cord compression in MRI images, along with deterioration or not-improving neurological status (27). Similarly, Rouanet et al. highlight that operative management is only indicated in substantial compression of the spinal cord with deterioration of neurological deficits (7). A2018 study by Wagner et al. favored surgery for patients with spinal column instability or persistent compression of the spinal cord after traumatic CCS without any neurologic improvement (Level B of recommendation) (4). Some studies claim that in patients with intact spinal canal, conservative treatment is preferred, regardless of the neurologic status, and only in neurologic deterioration cases isa prompt decompression warranted (4, 28). However, in a study by Qin et al. comparing conservative treatment and early and delayed surgery in patients with acute traumatic central cord syndrome without fracture or dislocation, no significant difference in neurological recovery was reported among the therapeutic approaches (29). Only when early decompression is performed, patients were found to experience faster motor function recovery (29). Similarly, another study by Khorasanizadeh et al. showed that neurologic deficit improvement is independent of the treatment choice-conservative versus surgical-but noted that the mechanism and characteristics of injury may affect the outcome (30). Some studies support that even in cases where patients were treated conservatively at first with continuous neurological improvement, delayed surgery may still be beneficial (4). In any case, the improvement of neurologic deficits post-operatively seems unpredictable (4).

Studies arguing against surgery focus not only on negative outcome results but also on the inherent risks of surgery. Mazaki et al. state that there is no significant difference in outcome comparing laminoplasty to conservative treatment, with surgery being associated with a higher frequency of complications (9). In their review, Atesok et al. refer that for SCIWORA patients with normal or pure intraneural MRI findings surgery is not recommended, no matter their neurological status (27). Possible complications of spinal canal decompression may be dural tear and subsequent CSF-leakage, nerve root palsy, wound infection, CNS infection, pneumonia and respiratory failure, thromboembolic events, cardiac arrest, or death (4, 20, 31). The complication and morbidity rates should be taken into account together with the previously mentioned negative results (i.e., results showing no benefit of surgery over conservative treatment) when choosing the optimal treatment for a patient, especially when treating older patients or patients with comorbidities.

Although we did include SCWIORA in our search algorithm, we tried to avoid the term in our manuscript for several reasons: Firstly, we feel that the term itself is rather outdated; it refers mainly to the absence of radiographic abnormality (no radiographic or computed tomographic features of spinal fracture or instability as per the original paper by Paul and Wilberger) and in the modern era of widespread MRI-availability it is rather unusual to have clinical signs of spinal cord trauma without any radiographic evidence of injury. Secondly, the term has a strong connotation to spinal injuries in children (32). Adults who have spinal cord injury without computed tomographic evidence of trauma have CT-scans showing canal stenosis and significant degenerative changes in the cervical spine; thus, it is not accurate to state that they have SCIWORA (33).

A limitation of this study is the limited number of eligible studies included and thus the restricted sample size for data extraction and result forming. This limits our ability to detect potential associations that may be statistically significant in our analysis. Moreover, the various types of surgical treatments used in each individual study were categorized into techniques of lamina- or dura- decompression in order to summarize our results. Most papers investigate cervical spinal cord injury and thus, studies for patients with thoracic or lumbar spine injuries are missing. Finally, it should be noted that the majority of the studied population consists of patients having additionally some degree of degenerative-type spinal stenosis (intervertebral disk, spondylosis, ossified posterior longitudinal ligament etc.), and thus there are no formal results regarding cases with fully uncompressed spinal canal.

In our review, the majority of studies found that decompression of the spinal canal improves post-operative ASIA grade. The amount of data, however, is scarce and one should be cautious to interpret these results as more than a trend favoring decompression after spinal cord injury, even in the absence of traumatic compromise of the spinal canal. Ultimately, the type of surgical approach may not be the defining factor, provided that the approach itself generates an environment within the spinal canal able to mitigate or alleviate secondary injury to the spinal cord.

## Conclusion

There is no clear consensus on the management of spinal cord injuries without apparent traumatic obstruction of spinal canal. Increasing evidence suggests that osseous decompression and restoration of the spinal alignment may not be sufficient to adequately address intraspinal pathogenetic cascades, thus leading surgeons to intervene by opening the dura and further releasing the spinal cord. The potential drawback of this approach is longer operative times and a higher complication rate. While in literature there seems to be a trend towards surgery, further research is necessary to more clearly define the surgical indications and evaluate the benefit of bony and dura decompression in neurological outcome.

## Data availability statement

The original contributions presented in the study are included in the article/Supplementary material, further inquiries can be directed to the corresponding author.

## Author contributions

MS and LS: conceptualization and formal Analysis. MS, LS, and EB: methodology. MS, LS, IY, AP, MG, EP, SB, and GS: investigation. LS: supervision. LS and MG: manuscript editing. All authors contributed to the article and approved the submitted version.

## Conflict of interest

The authors declare that the research was conducted in the absence of any commercial or financial relationships that could be construed as a potential conflict of interest.

## Publisher’s note

All claims expressed in this article are solely those of the authors and do not necessarily represent those of their affiliated organizations, or those of the publisher, the editors and the reviewers. Any product that may be evaluated in this article, or claim that may be made by its manufacturer, is not guaranteed or endorsed by the publisher.
