# Supplementary material for: Posterior spinal decompression in adults with spinal cord injury without traumatic compromise of the spinal canal: what is the data?
Source: Front Neurol. 2023 Sep 15;14:1220598. doi: 10.3389/fneur.2023.1220598 (PMC10543663; doi:10.3389/fneur.2023.1220598)
Supplement: Supplementary file 2 [file Table_2.pdf]

SUPPLEMENTARY MATERIAL 2

ALGORITHM of SEARCH
[<br><br>**SPINAL CORD INJURY** or **SCI** or **SPINAL CORD TRAUMA** or **CENTRAL CORD SYNDROME** or **SCIWORA** or **SPINAL CORD INJURY WITHOUT RADIOGRAPHIC ABNORMALITY**<br><br>AND<br><br>**CERVICAL** or **THORACIC** or **LUMBAR** or **THORACOLUMBAR**<br><br>AND<br><br>**LAMINECTOMY** or **LAMINOPLASTY** or **DUROPLASTY** or **DUROTOMY** or **MENINGOPLASTY** or **DECOMPRESSION**<br><br>]<br><br>AND<br><br>**English** (Languages)
